# Lingonberry (*Vaccinium vitis-idaea*) press-cake as a new processing aid during isolation of protein from herring (*Clupea harengus*) co-products

**DOI:** 10.1016/j.fochx.2023.100592

**Published:** 2023-02-04

**Authors:** Jingnan Zhang, Mehdi Abdollahi, Anna Ström, Ingrid Undeland

**Affiliations:** a Department of Biology and Biological Engineering- Food and Nutrition Science, Chalmers University of Technology, SE 412 96, Sweden; b Department of Chemistry and Chemical Engineering – Applied Chemistry, Chalmers University of Technology, SE 412 96, Sweden

**Keywords:** Protein recovery, Protein isolation, Fish, Lipid oxidation, Volatile aldehydes, Acid consumption, Side stream valorization

## Abstract

High acid-consumption and lipid oxidation are challenges when recovering functional proteins from herring co-products via pH-shift-processing. Here, lingonberry press-cake (LP), which is abundant in organic acids and phenolics, was added to alkali-solubilized herring-*co*-product-proteins (2.5–30 % LP per dry weight) aiming to aid protein precipitation, save hydrochloric acid (HCl) and provide oxidative stability. The results revealed 5–30 % LP addition reduced HCl-consumption by 13–61 % and 19–79 % when precipitating proteins at pH 5.5 and 6.5, respectively. Higher LP% decreased protein content and lightness of protein isolates but raised the lipid content. Precipitation at pH 6.5 used less acid, reduced total protein yield and raised moisture content and darkness of isolates. Contrary to controls, lipid oxidation-derived volatiles did not develop in protein isolates precipitated with 10 % and 30 % LP, neither during the process itself nor during 21 days on ice. Altogether, LP was identified as a promising all-natural processing-aid to use during herring protein isolation.

## Highlights

• Lingonberry presscake (LP) was added to aid precipitation in the pH-shift process.
• Adding LP reduced the hydrochloric acid consumption up to 79%.
• Adding LP did not negatively affect the total protein yield.
• ≥10 % LP prevented lipid oxidation of protein isolates during 21 days on ice.
• Precipitation with 10% LP was considered most promising.

## Graphical abstract

**Figure:**
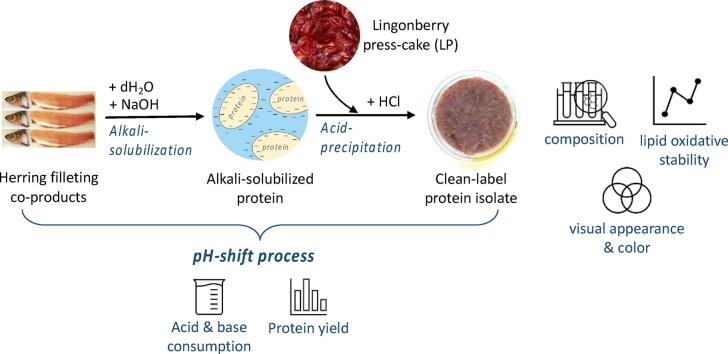

## 1 Introduction

Herring (*Clupea harengus*) is a small, dark, muscle-rich fish that yielded the fourth-highest capture production among all finfish species in 2018 (FAO, 2020), reaching 1820 metric tons of live weight (FAO, 2020). Herring is highly suitable for human consumption due to its abundance of high-quality protein, long-chain (LC) n-3 polyunsaturated fatty acids (PUFAs), vitamins (*e.g.*, D and B12) and minerals (Larsen et al., 2011). However, after the filleting process, 40–60 % of the herring (wet weight basis) ends up as co-products, which normally leave the food chain (Wu et al., 2021). It has been previously shown that the heads and frames are promising sources of functional proteins that can be recovered by pH-shift processing, *i.e.,* acid or alkaline protein solubilization followed by isoelectric precipitation (Park, 2013). The produced protein isolates are suitable for food production due to their high nutritional values and techno-functionalities *e.g.,* their ability to form a gel, foam and emulsion (Park, 2013). Protein isolate-derived gels can be successfully converted to food products *e.g.*, crab sticks, burgers and kamaboko (Park, 2013). However, the pH-shift process consumes significant amounts of acid and alkali, usually HCl and NaOH, which increases cost and results in the formation of NaCl (Marmon, 2012). Another potential downside is that when raw materials are high in hemeproteins, the development of lipid oxidation products has been documented (Wu et al., 2021, Zhang et al., 2022). Adding lingonberry press-cake (LP) to herring filleting co-products at the start of the pH-shift process was recently proven to prevent oxidation of the herring lipids both during the process itself and during subsequent storage of the recovered protein isolates (Zhang et al., 2022). In addition, this strategy was proven to be generally favorable in a life cycle assessment (LCA) considering climate change, cumulative energy demand, land occupation, and depleted stock fraction impacts of marine resources (Coelho et al., 2022, Coelho et al., 2023). However, a drawback of this strategy was that the consumption of base and acid increased since organic acids of LP reduced the pH of the raw starting material (Abdollahi et al., 2020; Kylli et al., 2011). Additionally, the protein yield of the process was reduced, possibly due to protein–polyphenol interactions hampering solubilization (Zhang et al., 2022). To counteract these problems but still take advantage of the outstanding antioxidant capacity of LP, we hypothesized that the LP-aided pH-shift processing concept could be significantly improved by adding LP in the protein precipitation step of the pH-shift process instead of at the start. The organic acids and polyphenols of LP could then aid the precipitation of alkali-solubilized herring proteins, thereby decreasing HCl usage at the same time that lipid oxidation product formation is minimized. To the best of our knowledge, using a plant-based food co-product as a precipitation aid during pH-shift-based muscle protein isolation has not been reported before. If successful, this new cross-processing concept could pave the way for industrial symbiosis which adds value to underutilized or wasted marine and agricultural side streams.

The specific aims of the present study were to investigate how much the HCl demand decreased when adding 2.5–30 % (dry weight (dw)/dw) LP during precipitation of alkali-solubilized herring co-product proteins at pH 5.5 or 6.5 and to monitor whether the antioxidative capacity was maintained when adding the LP at the second step of the pH-shift process rather than at the start. The impacts of LP addition and different precipitation pH levels (5.5 *vs* 6.5) on protein solubility, protein yield and characteristics of protein isolates (color and proximate composition) were also studied.

## 2 Materials and methods

### 2.1 Preparation of herring and lingonberry raw materials

Fresh heads and backbones from herring (*Clupea harengus*) (Sweden Pelagic, Ellös, Sweden) were packed separately and directly transported on ice to Chalmers University of Technology in October 2019. Herring heads and backbones were manually mixed in a 1:1 ratio and ground in a tabletop meat grinder with a 4.5 mm hole plate (C/E22 N; Minerva Omega, Bologna, Italy). The mince was packed in plastic bags and stored at −80 °C. The laboratory-produced lingonberry (*Vaccinium vitis-idaea*) press-cakes (LLPs) were prepared from frozen lingonberries that were purchased from a local food store. The frozen lingonberries were defrosted, mixed with a hand blender (5KHB1231; KitchenAid, St. Joseph, MI, USA) and centrifuged (Sorvall LYNX 6000; Thermo Fisher Scientific, Osterode am Harz, Germany) (5,000×*g*, 10 min, 10 °C). The resulting pellets were collected, packed in plastic bags and stored at −80 °C. The lingonberry (*Vaccinium vitis-idaea*) press-cakes from industrial juice production (ILPs) (Grangärde Musteri, Grangärde, Sweden) was transported to Chalmers University of Technology in November 2019 after being stored at −20 °C for five months. The ILP contained peels, seeds, leaves, stems and leftover flesh. On a wet weight basis, the amounts of leaves and seeds were 9 % and 24 %, respectively. The ILP was stored at −80 °C after being ground in a similar manner as the herring co-products.

### 2.2 Alkali-aided pH-shift processing of herring co-products without and with LLP/ILP

Alkali-aided pH-shift processing of co-products was conducted as described by Abdollahi et al. (2020). The frozen herring co-product mince was thawed in a sealed plastic bag under cold running water until the core temperature reached 0 °C. The herring co-products were mixed manually with 6 parts water (*i.e.*, 1:6 w/w). The mixture was homogenized (T18 digital Ultra-Turrax; IKA, Staufen, Germany) at 10,000 rpm for 60 s. The pH of the homogenate (H) was initially 6.8 ± 0.1. Then, the pH was adjusted to 11.5 by 2 M NaOH for protein solubilization. After incubating on ice for 15 min, the homogenate was centrifuged (8500×*g*, 4 °C, 20 min) which yielded three phases: a floating lipid layer, a supernatant (S1) containing alkali-solubilized proteins, and an insoluble sediment at the bottom. The lipid layer and the sediment were removed by using sieves, and S1 was collected. LLP was added to S1 at this stage in different amounts (corresponding to 0, 2.5 %, 5 %, 10 %, 20 % and 30 % of the dry weight of the herring co-products). ILP was added to S1 in two amounts (10 % and 30 %, dw/dw) (see amount of LP addition Supplementary Table 1). The mixture of S1 and LLP was homogenized (10,000 rpm, 60 s), and the pH of the homogenate was then recorded and adjusted to 6.5 and 5.5 by adding 2 M HCl. The volume of HCl that was used was also recorded. The homogenate was then incubated on ice for 15 min and centrifuged (8,500 × g, 4 °C, 20 min). Two phases were obtained: the supernatant (S2) and the pellet consisting of precipitated protein isolate. S2 was removed by a sieve and the protein isolates were packed in sealed plastic bags and stored at −80 °C. The entire pH-shift process was conducted on ice. The alkali-aided pH-shift processing of herring co-products with and without LLP/ILP was conducted in duplicate (*n_e_* = 2). H, S1 and S2 were collected for protein measurements using the method by Markwell et al. (1978). Protein solubility and protein yield were determined based on duplicate or triplicate subsamples (n ≥ 2) of each fraction using Eq. (1)-(5), where $c_{\;x}$ and $V_{x}$ are the protein concentrations and the volumes, respectively.

Protein solubility (${\mathrm{Solubility}}_{1}$) and protein yield (${\mathrm{Yield}}_{1}$) during the protein solubilization step were calculated as follows:

(1) $${\mathrm{Solubility}}_{1}\left(\%\right)\;=\frac{c_{\;S1}}{c_{\;H}}\times 100$$

(2) $${\mathrm{Yield}}_{1}\left(\%\right)=\frac{c_{\;S1}\times V_{S1}}{c_{\;H\times }V_{H}}\times 100$$

Protein solubility (${\mathrm{Solubility}}_{2}$) and protein yield (${\mathrm{Yield}}_{2}$) during protein precipitation step were calculated as follows:

(3) $${\mathrm{Solubility}}_{2}\left(\%\right)=\frac{c_{\;S2}}{c_{\;S1}}\times 100$$

(4) $${\mathrm{Yield}}_{2}\left(\%\right)=\left(1-\frac{c_{S2}\times V_{S2}}{c_{S1}\times V_{S1}}\right)\times 100$$

The total protein yield (Totalyield) over the entire process was calculated as follows:

(5) $$\mathrm{Totalyield}\left(\%\right)=\frac{c_{S1}\times V_{S1}-c_{S2}\times V_{S2}}{c_{\;H\times }V_{H}}\times 100$$

### 2.3 Ice storage of protein isolates

Protein isolates produced without and with ILP were adjusted to a moisture content of 80 % and a pH of 7 before being subjected to the 21-day ice storage trial which was conducted as described by Zhang et al. (2022). Streptomycin (200 ppm on a moisture basis) was manually stirred into samples to prevent microbial growth. Each sample type was then stored as a thin layer (5–6 mm thick) at the bottom of duplicate 250 mL Erlenmeyer flasks (n = 2). The caps of the flasks were screwed tightly, and the whole flask was wrapped in aluminum film to avoid light. The flasks were placed on ice in insulated cooler boxes in a 4 °C cold room, and ∼ 0.7 g of sample was taken out regularly during ice storage for chemical analyses of lipid oxidation. The samples were also subjected to a daily sensorial screening for rancid odor using a scale from 0 to 100, following the method described by Sannaveerappa et al. (2007) (see rancid odor results in Supplementary Fig. 1).

### 2.4 Proximate composition analysis

The moisture and ash content of protein isolates were determined by placing the samples at 105℃ and 550℃, respectively (AOAC, 2005). The crude protein content of herring co-products, LP, and protein isolates was measured by a modified version of the Dumas method with a LECO nitrogen analyzer (TruMac-N; LECO, St. Joseph, MI, USA), as described by Marcó et al. (2002). A nitrogen-to-protein conversion factor of 5.58 was used for herring co-products and protein isolates (Mariotti et al., 2008), and 5.4 was used for LP (Abdollahi et al., 2020). The crude lipid content was analyzed gravimetrically by a modified version of Lee’s method using extraction with chloroform and methanol (Zhang et al., 2022). Compositional measurements were performed on at least triplicate subsamples from each isolate type (n ≥ 3).

### 2.5 Color measurement of fresh protein isolates

Three grams of protein isolate was placed in a small petri dish and flattened into a 5–6 mm layer, after which the dishes were stored with lids in the same way as the E-flasks. During ice storage, the surface color was measured (n ≥ 3) in the CIE *L^∗^a^∗^b^∗^* color space using a colorimeter (CR-400; Konica Minolta, Osaka, Japan) after removing the lids (Abdollahi et al., 2020).

### 2.6 Analysis of volatile aldehydes formed during ice storage of protein isolates

Protein isolate samples (∼0.7 g) taken during storage trials were thawed in a tight plastic bag under cold running water. Samples were then weighed and manually squeezed with 4 mL of Milli Q water against the walls of a glass test tube using a Potter-Elvehjem piston. The homogenate was transferred to a 20 mL SPME vial whereafter the glass tube and pestle were rinsed with 4 mL of Milli-Q water, which was combined with the first 4 mL in the vial. Two hundred microliters of internal standard (2.5 mM 3-Methyl-3-buten-1-ol in Milli-Q water) was added, followed by vortexing for 10 s. The volatile compounds were analyzed via headspace solid-phase microextraction (HS-SPME) coupled with gas chromatography-mass spectrometry (GC–MS) according to the method described by Sajib et al. (2020). In brief, the extraction of volatiles was conducted for 20 min at 60℃ under stirring (500 rpm) after equilibration at 60℃ for 5 min with stirring. A 75 μm Carboxen/polydimethylsiloxane (CAR/PDMS)-coated SPME fiber (Supelco, Bellefonte, PA, USA) was used to adsorb the volatiles. The GC–MS analysis consisted of desorption of volatiles for 10 min at 300℃, followed by separation using a fused silica ZB-1701 capillary column (30 m × 0.32 mm, 1 μm) (Phenomenex, Torrance, CA, USA). The MS was operated in electron ionization mode and ions were scanned in the range of 10–250 amu. Hexanal, (E)-2-hexanal, heptanal, octanal and 2,4-heptadienal were selected as lipid oxidation markers since they were among the most abundant volatiles detected and since they are common to oxidized fish and other samples rich in n-3 or n-6 PUFAs (Aitta et al., 2021, Kakko et al., 2022). The retention time and SIM mass for identification of the volatile aldehydes used as lipid oxidation markers are presented in Supplementary Table 2.

### 2.7 Total phenolic content (TPC) analysis

The extraction of phenolic compounds was performed with 70 % methanol containing 1 % trifluoroacetic acid as described by Zhang et al. (2022). TPC was measured by a modified version of the Folin-Ciocalteu colorimetric method (Ainsworth & Gillespie, 2007) and was expressed as gallic acid equivalents (GAE) (g/100 g sample, dw).

### 2.8 Anthocyanin profile analysis

Anthocyanin extraction was performed with methanol acidified by 0.3 % HCl (v/v) as described by Bunea et al., 2013, You et al., 2011 with minor changes. In brief, 3 mL of acidified methanol was added to 0.2 g of freeze-dried ILP in a glass tube. Nitrogen gas was used to flush out the air from the tube. After vortexing for 10 s, the samples were placed in the dark for 18 h, followed by sonication (S15; Elma Schmidbauer, Singen, Germany) for 15 min at 20 °C using 37 kHz. The sonicated samples were centrifuged (2000×*g*, 10 min) and the supernatants were collected. re-extraction was carried out by adding 3 mL of acidified methanol to the pellet, followed by vortexing and centrifugation. The supernatant from re-extraction was combined with the first extraction. The combined supernatant, 6 mL in total, was centrifuged at 4000×*g* for 15 min and stored at −20 ℃ until analysis. Anthocyanin profiles were determined using the high-performance liquid chromatogram (HPLC)-ultraviolet/visible (UV/VIS) method as described by Oliveira et al. (2019). The system consisted of a quaternary gradient pump (PU-2089 Plus; Jasco, Easton, MD, USA), a cooled (8 °C) autosampler (AS-2057 Plus; Jasco, Easton, MD, USA), and a UV–vis detector (SPD-10A; Shimadzu, Kyoto, Japan).

### 2.9 Statistical analysis

Significant differences between sample groups were determined by one-way analysis of variance (ANOVA) followed by Duncan’s multiple range test by using SPSS Statistics (version 27; IBM, New York, NY, USA). The significance level (p) was set at 0.05, below which the differences were considered significant.

## 3 Results and discussion

### 3.1 LP-induced pH changes of the first supernatant (S1) and required volumes of acid to reach the precipitation pH

**Fig. 1:**
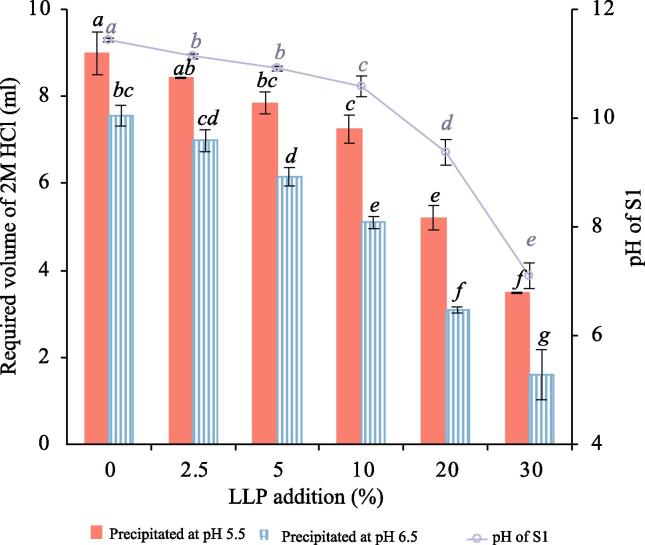
The required volume of 2 M HCl to adjust the first supernatant from the alkali-aided protein solubilization process (S1) to pH 5.5 or 6.5 when processing 100 g herring co-products (**bars**) and the starting pH of S1 (**line**) without and with adding 2.5–30 % (dw/dw) LLP (LLP = Lab produced lingonberry press cake) (bars). Data shown are average values ± standard deviation (*n_e_* = 2). Data with different superscript letters are significantly different (p < 0.05).

As shown in Fig. 1, the pH of S1 after the first centrifugation step was 11.4 ± 0.0, but with increasing addition of LLP this value decreased, reaching 7.1 ± 0.2 after 30 % addition (dw/dw). This can be explained by the abundance of organic acids in LLP such as benzoic acid, citric acid, malic acid (Klavins et al., 2021, Mikulic-Petkovsek et al., 2012, Viljakainen et al., 2010), cinnamic acid (Klavins et al., 2021), tartaric acid, fumaric acid and shikimic acid (Mikulic-Petkovsek et al., 2012), providing it with a low pH (pH = 2.9). Additionally, in our previous study, we found that adding 30 % (dw/dw) LLP to herring or salmon co-products at the start of the protein solubilization step significantly decreased the pH of the raw material-in-water homogenate from 6.9 to 5.0 (herring) and from 6.2 to 4.7 (salmon) (Abdollahi et al., 2020). In the present study, when processing 100 g of herring co-products in the absence of LLP, ∼9 mL of 2 M HCl was required to adjust the pH of S1 from 11.5 to 5.5, which is the herring protein pI (Abdollahi and Undeland, 2018, Geirsdottir et al., 2007, Zhang et al., 2023). However, adding 5–30 % LLP to S1 significantly reduced the requirement for 2 M HCl to reach pH 5.5. Compared to the control without LLP, adding 5 %, 10 %, 20 % and 30 % LLP to S1 reduced the consumption of acid by 13 %, 20 %, 42 % and 61 %, respectively. As a strategy to further reduce acid consumption and avoid acidic pH conditions, which are known to stimulate Hb-mediated lipid oxidation (Abdollahi et al., 2016), pH 6.5 was used as an alternative protein precipitation pH. Going from pH 11.5 to 6.5 required ∼7.6 mL of 2 M HCl per 100 g of processed herring co-products, which was significantly reduced by 19 %, 33 %, 59 % and 79 % when adding 5 %, 10 %, 20 % and 30 % LLP, respectively. Overall, the consumption of HCl was significantly (p < 0.05) reduced by 16 %, 17 %, 22 %, 30 %, 41 % and 54 % when precipitating proteins at pH 6.5 instead of pH 5.5 without ILP and in the absence or presence of 2.5 %, 5 %, 10 %, 20 % and 30 % LLP, respectively (Fig. 1).

### 3.2 Protein solubility and protein yield during pH-shift processing when using LLP-aided precipitation

The protein solubility and protein yield measured during the alkali protein solubilization step of the pH-shift process were 86.7 ± 0.9 % and 73.7 ± 0.8 %, respectively. When using pH 6.5 as the precipitation pH rather than pH 5.5, the protein solubility was significantly higher, and the protein yield was significantly lower (Table 1). The difference between pH 6.5 and 5.5 was 5–7 % for protein solubility and 4 % for protein yield because pH 6.5 is one pH-unit away from the herring protein pI (pH 5.5) (Abdollahi and Undeland, 2018, Geirsdottir et al., 2007). The addition of LLP showed no significant impacts on protein solubility or protein yield in the precipitation step using pH 5.5 or pH 6.5 or the total protein yield of the entire process (Table 1). This finding showed that adding LLP in the precipitation step is more promising than adding LLP at the start of the process; the latter had a significant reduction in total protein yield, which was reported in our previous study (Abdollahi et al., 2020).

**Table 1:** Protein solubility and protein yield measured during the precipitation step (*i.e.*, the step in which pH was adjusted to pH 5.5/6.5 followed by a second centrifugation) and total protein yield when cross-processing herring co-products without/with LLP-aided precipitation at addition ratios from 2.5 to 30 % (dw/dw). Data are given as percentages showing mean values ± standard deviation (*n_e_* = 2, n ≥ 2).

Precipitation pH	LLP addition (%)	Protein solubility in precipitation step (%)	Protein yield in precipitation step (%)	Total protein yield (%)
5.5	0	9.8 ± 0.5^b^	91.4 ± 0.4^a^	67.7 ± 0.2^a^
	2.5	10.5 ± 0.0^b^	91.0 ± 0.0^a^	67.3 ± 0.5^a^
	5	10.9 ± 0.1^b^	90.8 ± 0.3^a^	67.2 ± 0.3^a^
	10	10.7 ± 0.4^b^	91.1 ± 0.4^a^	67.4 ± 0.1^a^
	20	10.6 ± 0.3^b^	91.2 ± 0.3^a^	67.5 ± 0.7^a^
	30	10.5 ± 0.3^b^	91.8 ± 0.3^a^	67.9 ± 0.7^a^
6.5	0	14.6 ± 1.5^a^	87.7 ± 1.2^b^	64.3 ± 0.1^b^
	2.5	14.9 ± 0.6^a^	87.3 ± 0.5^b^	64.1 ± 0.7^b^
	5	15.5 ± 1.3^a^	87.0 ± 1.1^b^	63.8 ± 0.2^b^
	10	15.6 ± 1.8^a^	87.2 ± 1.4^b^	63.9 ± 0.0^b^
	20	16.0 ± 0.8^a^	87.3 ± 0.5^b^	64.0 ± 0.7^b^
	30	17.2 ± 1.5^a^	87.6 ± 1.0^b^	64.3 ± 0.3^b^

Data within the same column carrying a different superscript letter are significantly different (p < 0.05).

LLP = Lab produced lingonberry press cake.

### 3.3 Proximate composition of raw materials and protein isolates produced with LLP and ILP

There was a significant increase in the concentration of protein during the pH-shift processing of herring co-products, both without and with LP (Table 2), which confirmed the ability of this process to partition lipids and ash into the floating layer and first sediment, respectively. This gravity-based separation of oil, protein and ash is driven by the different densities of these fractions and is in line with previous studies of the pH-shift process (Chomnawang and Yongsawatdigul, 2013, Panpipat and Chaijan, 2017, Singh et al., 2019). As expected, the composition of the produced protein isolates was affected by the addition ratio and type of LP, as well as the precipitation pH used during the pH-shift process. The moisture content of protein isolates ranged from 86 to 93 g/100 g, which is representative of herring protein isolates produced by pH-shift processing (Abdollahi et al., 2020). For protein isolates precipitated at the same pH, their moisture content increased with an increasing ratio of added LP, indicating an increasing water-holding capacity of the cross-process protein isolates. Increasing the precipitation pH from pH 5.5 to pH 6.5 also significantly increased the moisture content of protein isolates, which was due to the higher negative net charge of the proteins further away from the pI (Belitz et al., 2009). Compared to LLP, ILP gave significantly lower moisture contents of protein isolates, which was due to the significantly lower moisture content of ILP than LLP. This difference translated into a need to add more LLP to S1 to reach a fixed ratio between the herring co-products' dry weight and the LP dry weight, thereby adding more water to the system. Table 2 also shows that the protein content of the protein isolates decreased with increasing LP addition, which was an expected result of diluting the herring proteins by a high carbohydrate raw material such as LP having only 3.4–5.6 g protein/100 g dw. ILP, which was more lipid-rich than LLP, yielded significantly lower protein content and higher lipid content of protein isolates than LLP when used at the same levels (10 and 30 %). Overall, the relatively high lipid content of the two LPs translated into protein isolates with significantly higher lipid content as more LP that was added. This was different from the ash content of the protein isolates which was only significantly increased at 20 % and 30 % LP addition (dw/dw), respectively. This could be explained by the fact that the ash brought in by the LP was counteracted by a decreased usage of 2 M HCl (Fig. 1), which subsequently created less NaCl. The TCC of LLP and ILP was 84.3 ± 1.1 and 78.8 ± 0.7 d-glucose equivalents (GE) (g/100 g, dw), respectively. The estimated content of carbohydrates in the two LPs based on data on protein, lipid and ash contents was 85 g/100 g dw and 78 g/100 g dw for LLP and ILP, respectively. The carbohydrate content of the protein isolates thus increased when increasing the LP addition from 2.5 % to 30 % (dw/dw).

**Table 2:** Proximate composition of herring co-products, LLP, ILP and protein isolates produced thereof. Protein, lipid, and ash data are given as g/100 g dw with data showing mean values ± standard deviation (n ≥ 3).

		LP addition (%)	Precipitation pH	Moisture (% ww)	Protein (% dw)	Lipid (% dw)	Ash (% dw)
Herring co-products				71.0 ± 0.2^k^	48.5 ± 0.8^j^	32.7 ± 1.5^a^	13.4 ± 0.1^a^
LLP				79.2 ± 0.5^i^	3.4 ± 0.1^k^	10.0 ± 0.5^j^	1.4 ± 0.1^g^
ILP				75.6 ± 0.1^j^	5.6 ± 0.1^k^	14.4 ± 2.1^i^	2.0 ± 0.1^f^
Protein isolates	+ LLP	0	5.5	85.9 ± 0.2^h^	83.0 ± 1.0^a^	13.7 ± 0.3^i^	3.0 ± 0.1^e^
			6.5	89.2 ± 0.1^e^	80.1 ± 1.3^bc^	14.3 ± 0.3^gh^	3.0 ± 0.3^e^
		2.5	5.5	86.3 ± 0.1^gh^	82.0 ± 0.2^ab^	15.8 ± 1.1^i^	3.0 ± 0.1^e^
			6.5	89.4 ± 0.1^e^	78.4 ± 1.9^c^	17.1 ± 0.4^fg^	3.1 ± 0.2^e^
		5	5.5	86.9 ± 0.4^g^	78.4 ± 3.4^c^	18.1 ± 0.8^h^	3.0 ± 0.3^e^
			6.5	90.0 ± 0.5^d^	73.6 ± 0.2^d^	19.0 ± 1.6^ef^	3.0 ± 0.2^e^
		10	5.5	87.8 ± 0.2^f^	72.4 ± 3.0^de^	16.5 ± 0.4^fg^	3.0 ± 0.1^e^
			6.5	90.8 ± 0.5^c^	68.8 ± 0.5^f^	17.4 ± 0.4^e^	3.2 ± 0.2^de^
		20	5.5	88.3 ± 0.3^f^	70.5 ± 0.6^ef^	18.4 ± 0.1^ef^	3.1 ± 0.1^e^
			6.5	91.6 ± 0.3^b^	66.3 ± 1.6^g^	18.8 ± 0.3^cde^	3.4 ± 0.1^cd^
		30	5.5	90.1 ± 0.4^d^	62.8 ± 2.1^h^	19.4 ± 0.7^de^	3.4 ± 0.0^cd^
			6.5	92.9 ± 0.6^a^	60.2 ± 1.0^i^	20.4 ± 0.5^cd^	4.0 ± 0.2^b^
	+ ILP	10	5.5	86.7 ± 0.3^g^	64.4 ± 1.6^gh^	20.7 ± 0.5^c^	3.0 ± 0.1^e^
		30	5.5	87.8 ± 0.1^f^	50.4 ± 0.9^j^	24.5 ± 0.9^b^	3.6 ± 0.1^c^

Data within the same column carrying a different superscript letter are significantly different (p < 0.05).

LLP = Lab produced lingonberry press cake, ILP = Industrially produced lingonberry press cake.

### 3.4 Visual appearance and color of protein isolates produced with LLP

**Fig. 2:**
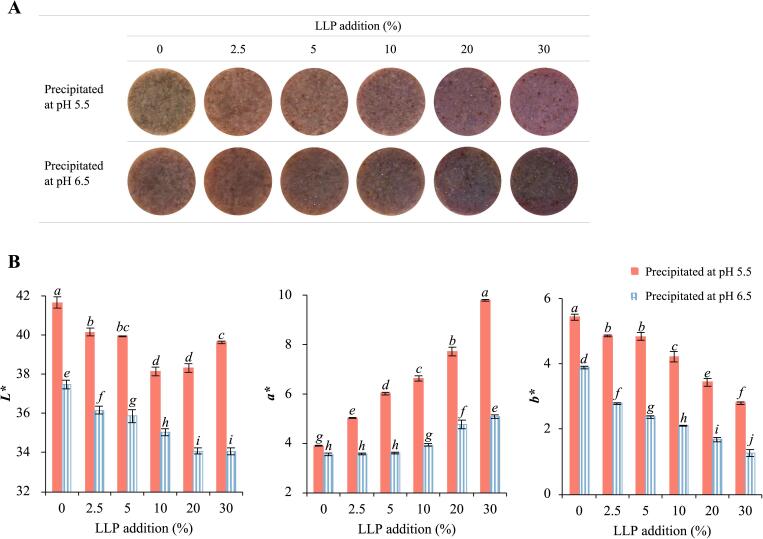
Visual appearance (**A**) and color attributes using the L*a*b* color space (**B**) of protein isolates precipitated at pH 5.5 or pH 6.5 with increasing addition of LLP. Data shown are average values ± standard deviation (n = 3). Data with different superscript letters are significantly different (p < 0.05). LLP = Lab produced lingonberry press cake.

As expected, the addition of LLP to the alkali-solubilized herring proteins (S1) changed its color from red to purple; and thereby also changed the visual appearance of the produced protein isolates (Fig. 2**A**). However, as shown in Fig. 2**B**, the surface color of protein isolates was impacted by both the precipitation pH (5.5 or 6.5) and the LLP addition ratio. Protein isolates precipitated at pH 5.5 were visually lighter than those precipitated at pH 6.5, which was supported by significantly increased L*-values. This was most likely related to the anthocyanins found in lingonberry (Rein & Heinonen, 2004), which are known to change from bright reddish to darker purple as they gradually become deprotonated when going from low pH (1–3) to higher pH (6–7) (Fossen et al., 1998). With increasing LLP additions, the L* values of the protein isolates precipitated at pH 6.5 decreased, while the L* values of the protein isolates precipitated at pH 5.5 decreased only until 10 % LLP was added (dw/dw). The latter was followed by an increase in L* between 10 % and 30 % LLP addition which could be explained by the significantly increased moisture contents (Table 2). Higher moisture dilutes the pigments but also causes increased light reflection (Abdollahi et al., 2020). An increasing LLP addition ratio also significantly increased a*- values (Fig. 2**B**), which could be explained both by the increased amount of LLP-derived anthocyanins in the system (Rein & Heinonen, 2004) and the strong antioxidant capacity of LLP (Abdollahi et al., 2020, Zhang et al., 2022). The latter could prevent the oxidation of hemeproteins into their brown met-forms which otherwise takes place at slightly acidic pH values (Aranda et al., 2009, Wu et al., 2021). LLP pieces remained in the protein isolates since they were not soluble in the supernatant (S2) that formed during the second centrifugation. However, the LLP pieces were partitioned to the bottom of the sedimented protein isolate when using the current centrifugation conditions (8500×*g*, 20 min, at 4 °C). By optimizing the centrifugation conditions and by removing the bottom part of the protein sediment, it might thus be possible to produce protein isolates without LLP pieces. Whether it is an advantage or not to have a herring protein isolate containing LLP pieces will depend on the future application of such protein isolates in food products.

### 3.5 Volatile aldehydes derived from lipid oxidation during the ice storage of protein isolates

**Fig. 3:**
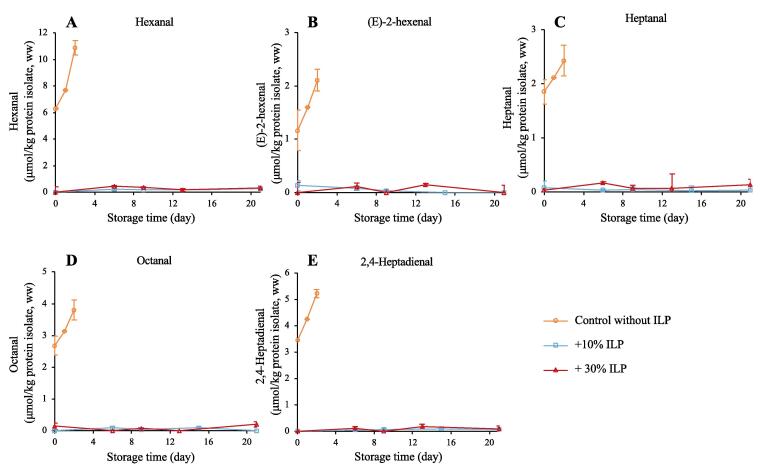
Hexanal (**A**), (E)-2-hexenal (**B**), heptanal (**C**), octanal (**D**) and 2,4-heptadienal (**E**) measured during ice storage of protein isolates produced without and with 10 % or 30 % ILP addition in the protein precipitation step. Volatile compounds were only measured up to the point when the rancid odor of the samples had peaked and started to decline, explaining why controls were only followed for 2 days. Sensorial screening was performed according to Sannaveerappa et al. (2007).

The formation of hexanal, (E)-2-hexenal, heptanal, octanal and 2,4-heptadienal, all of which are breakdown products of lipid hydroperoxides (Aitta et al., 2021, Kakko et al., 2022), was monitored during the 21-day ice storage of protein isolates. Hexanal can be produced by beta scission of the lipid hydroperoxide on either side of the radical and is primarily associated with the oxidation of n-6 PUFAs (Gómez-Cortés et al., 2015, Nogueira et al., 2019). Hexanal can however also be formed during the degradation of other preformed volatiles *e.g.*, 2-octenal and 2,4-decadienal (Iglesias & Medina, 2008). (E)-2-hexenal and 2,4-heptadienal are mainly produced from n-3 PUFAs (Domínguez et al., 2019, Pennarun et al., 2002). As shown in Fig. 3, all mentioned aldehydes were detected at elevated levels in the control sample at Day 0, *i.e.*, right after production of the isolate, and then continuously increased until Day 3 when sampling was stopped due to intense rancid odor. This confirms our previous findings that high levels of lipid oxidation products, lipid hydroperoxides, total carbonyls, malondialdehyde (MDA), 4-hydroxy-(E)-2-hexenal (HHE) and 4-hydroxy-(E)-2-nonenal (HNE), develop in herring co-products during the pH-shift method (Abdollahi et al., 2020, Zhang et al., 2023). This could be ascribed to the high-speed mixing of raw material in water at the start of the process, which diluted endogenous aqueous antioxidants such as ascorbic acid (Harrysson et al., 2020) and increased the exposure of membrane phospholipids to pro-oxidative Hb since the highly organized fish muscle microstructure is disrupted along with the removal of endomysium (Marmon, 2012). Second, the membrane-bound antioxidant α-tocopherol can be partly removed along with the removal of membranes during the first centrifugation step (Wu et al., 2021). Third, the low precipitation pH (5.5) may induce Hb deoxygenation and metHb formation, which increases the prooxidative activity of Hb via heme group exposure and hemin loss, respectively (Kristinsson and Hultin, 2004, Wu et al., 2021). The low pH may also increase the proximity between Hb/hemin and phospholipids because sarcoplasmic proteins such as Hb can coprecipitate with myofibrils and/or membranes around the protein pI (Abdollahi et al., 2016). However, none of the monitored aldehydes were detected in protein isolates precipitated with 10 or 30 % ILP addition throughout the entire 21 days of ice storage. This finding indicated that the antioxidant capacity of ILP was further promoted when ILP was added in the precipitation step compared to when ILP was added at the beginning of the pH-shift process (Zhang et al., n.d., Zhang et al., 2022). The latter strategy prevented the formation of the abovementioned volatile aldehydes for ∼8 and ∼15 days on ice, respectively, at ILP-levels of 10 and 30 % (dw/dw) (Zhang et al., n.d.). The TPC of ILP was 3.1 ± 0.0 g gallic acid equivalents (GAE)/100 g, dw. When 10 % or 30 % ILP was added during the precipitation step, the TPC of the produced protein isolates was 1.2 ± 0.1 and 2.4 ± 0.0 g GAE/100g, dw, respectively, which is significantly higher than the TPC-levels reached when adding 30 % ILP at the start of the pH-shift process (0.8 g GAE/100 g, dw) (Zhang et al., 2022). Indeed, when added at the start, only alkali-soluble antioxidants of the ILP were carried over to the isolate, among which ideain and procyanidin A1 were recently identified to be the most potent (Lei et al., n.d.). However, when added in the precipitation step, most of the ILP-derived antioxidants remained in the protein isolates, including those bound to the peels and seeds. In agreement with our results, 3 % (w/w) dried industrial lingonberry press-cake added directly to minced, deskinned herring fillets considerably decreased the formation of volatile oxidation products during 10 months of frozen storage (Damerau et al., 2020). The authors ascribed the antioxidant ability of their industrial lingonberry press-cake to its proanthocyanidins and flavan-3-ols (Damerau et al., 2020, Tian et al., 2018). The anthocyanin profile of the presently used ILP, which had a berry:leaf ratio of approximately 91:9 (ww) is shown in Supplementary Table 3**.** The most abundant anthocyanin was ideain, followed by keracyanin, tulipanin and malvin. Keracyanin was reported as a primary phenolic antioxidant in black raspberry when testing antioxidant capacity by using assays of ferric reducing antioxidant power (FRAP), 2,2′-azinobis (3-ethylbenzothiazoline-6-sulfonic acid) (ABTS), and 2,2-diphenyl-1-picrylhydrazyl (DPPH) (Tulio et al., 2008). Tulipanin is an abundant anthocyanin in black currant (Nour et al., 2013) and showed good antioxidant ability in DPPH and FRAP and β-carotene bleaching assays (Yang et al., 2019). Malvin was also reported to have antioxidant capacity when tested in FRAP, DPPH, ABTS, *N*—*N*—dimethyl-p-phenyl-enediamine dihydrochloride (DMPD), superoxide anion (O_2_^●−^) and cupric ion reducing antioxidant capacity (CUPRAC) assays (Huyut et al., 2017).

## 4 Conclusions

Adding LP to alkali-solubilized herring co-product proteins reduced HCl consumption for protein precipitation at pH 5.5 by 61 % when adding 30 % LP. Increasing the precipitation pH to 6.5 decreased acid usage, reaching a 79 % reduction of HCl at 30 % LLP addition, but reduced the total protein yield and provided protein isolates with higher moisture and darker color. At a given precipitation pH, increasing LP% increased the a*-values and lipid and moisture contents of the protein isolates but decreased the protein contents. These features must be balanced against each other. Interestingly, the lipid oxidation-derived aldehydes, hexanal, (E)-2-hexenal, heptanal, octanal and 2,4-heptadienal were all completely prevented during 21 days of ice storage by 10 % and 30 % LP addition. Compared to our previous study, this work revealed that adding LP during the precipitation step was more promising in preventing lipid oxidation than adding it at the start of the pH-shift process, probably due to the higher and more diverse antioxidant content (consisting of both aqueous and lipophilic molecules) presented when LP was added during the precipitation step. The resulting protein isolates from each strategy should, however, be subject to further studies, *e.g.*, of sensorial profiles. In summary, the results of this study revealed that LP can play dual roles during fish protein precipitation, both replacing a large part of the hydrochloric acid needed and providing antioxidants to the produced protein isolates in a clean-label manner.

## Declaration of Competing Interest

The authors declare that they have no known competing financial interests or personal relationships that could have appeared to influence the work reported in this paper.

## Data Availability

Data will be made available on request.
