# Supplementary material for: Lingonberry (Vaccinium vitis-idaea) press-cake as a new processing aid during isolation of protein from herring (Clupea harengus) co-products
Source: Food Chem X. 2023 Feb 4;17:100592. doi: 10.1016/j.fochx.2023.100592 (PMC9941359; doi:10.1016/j.fochx.2023.100592)
Supplement: Supplementary data 1 [file mmc1.docx]

***Supplementary Material for***

**Lingonberry (Vaccinium vitis-idaea) press-cake as a new processing aid during isolation of protein from herring (Clupea harengus) co-products**

Jingnan Zhang^a*^, Mehdi Abdollahi^a^, Anna Ström^b^, Ingrid Undeland^a^

^a^ Department of Biology and Biological Engineering- Food and Nutrition Science, Chalmers University of Technology, SE 412 96, Sweden.

^b^ Department of Chemistry and Chemical Engineering- Applied Chemistry, Chalmers University of Technology, SE 412 96, Sweden.

Corresponding Author: ^*^J. Zhang: [jingnan.zhang@chalmers.se](mailto:jingnan.zhang@chalmers.se), +46 739 587 057.

**Supplementary Table 1.** The amount of herring co-products and lingonberry press-cake (LP) used during the cross-processing; LP was added in an amount that corresponded to 2.5%-30% of herring co-products’ dry weight (dw), cold distilled water was added in an amount that corresponded to 6 times of herring co-products’ wet weight; Ratio of LP to S1 on a wet weight basis. LLP=Lab produced lingonberry press cake, ILP=Industrially produced lingonberry press cake.

| Type of LP | LP addition (%, dw/dw) | Combination of raw materials and water (g) | | | Ratio of LP to S1 (%, ww/ww) |
| --- | --- | --- | --- | --- | --- |
|  |  | Herring co-products | Water | LP |  |
| LLP | 2.5 | 100 | 600 | 3.5 | 0.6 |
|  | 5 | 100 | 600 | 6.9 | 1.2 |
|  | 10 | 100 | 600 | 13.8 | 2.3 |
|  | 20 | 100 | 600 | 27.6 | 4.7 |
|  | 30 | 100 | 600 | 41.4 | 7.0 |
| ILP | 10 | 100 | 600 | 11.6 | 2.3 |
|  | 30 | 100 | 600 | 34.8 | 6.8 |

**Supplementary Figure 1.** Rancid odor detected during ice storage of protein isolates. LLP=Lab produced lingonberry press cake, ILP=Industrially produced lingonberry press cake.

**Supplementary Table 2.** Retention time (RT) and SIM ion mass for identification of volatile aldehydes used as the indicators of lipid oxidation. For SIM mass, the number in bold indicates the target ion mass and the rest are the reference ions.

| Compound name | RT (min) | SIM mass |
| --- | --- | --- |
| Hexanal | 13.28 | 44 + 56 |
| (E)-2-hexenal | 17.60 | 40 + 55 + 83 |
| Heptanal | 18.82 | 44 + 70 + 81 |
| Octanal | 21.97 | 43 + 56 + 84 |
| 2,4-Heptadienal | 23.10 | 40 + 53 + 81 + 110 |

**Supplementary Table 3.** Anthocyanin profile of the industrially produced lingonberry press cake (ILP). The results are presented in μmol/g dry weight, dw (mean values ± standard deviation (n=2)).

| Anthocyanin compound | Common name | Concentration (μmol/g, dw) |
| --- | --- | --- |
| Cyanidin-3-galatoside | Ideain | 671.67 ± 56.57 |
| Cyanidin-3-rutinoside | Keracyanin | 79.91 ± 8.53 |
| Delphinidin-3-rutinoside | Tulipanin | 44.26 ± 2.58 |
| Malvidin-3,5-diglucoside | Malvin | 39.92 ± 2.33 |
| Delphinidin-3-galactoside |  | 3.24 ± 0.71 |
| Pelargonidin-3-rutinoside |  | 2.77 ± 0.61 |
| Delphinidin 3-glucoside | Myrtillin | 0.54 ± 1.47 |
| Cyanidin |  | n.d. |
| Cyanidin-3,5-diglucoside | Cyanin | n.d. |
| Cyanidin-3-glucoside | Kuromanin | n.d. |
| Cyanidin-3-arabinose |  | n.d. |
| Delphinidin |  | n.d. |
| Malvidin |  | n.d. |
| Malvidin-3-galactoside |  | n.d. |
| Malvidin-3- glucoside | Oenin | n.d. |
| Pelargonidin |  | n.d. |
| Pelargonidin-3-glucoside | Callistephin | n.d. |
| Peonidin |  | n.d. |
| Peonidin-3-arabinoside |  | n.d. |
| Peonidin-3-galactoside |  | n.d. |

n.d. = Not-detected.
